# Influence of the Heme Nitric Oxide/Oxygen Binding Protein (H-NOX) on Cell Cycle Regulation in *Caulobacter crescentus*

**DOI:** 10.1016/j.mcpro.2023.100679

**Published:** 2023-11-17

**Authors:** Cameron Lee-Lopez, Md.Shariful Islam, Ady B. Meléndez, Erik T. Yukl

**Affiliations:** 1 Department of Chemistry and Biochemistry, New Mexico State University, Las Cruces, New Mexico, USA; 2 Department of Mathematics and Physics, North South University, Dhaka, Bangladesh

**Keywords:** H-NOX, *Caulobacter crescentus*, proteome, cell cycle, biofilm

## Abstract

The ability of an organism to respond to environmental changes is paramount to survival across a range of conditions. The bacterial heme nitric oxide/oxygen binding proteins (H-NOX) are a family of biofilm-regulating gas sensors that enable bacteria to respond accordingly to the cytotoxic molecule nitric oxide. By interacting with downstream signaling partners, H-NOX regulates the production of the bacterial secondary messenger cyclic diguanylate monophosphate (c-di-GMP) to influence biofilm formation. The aquatic organism *Caulobacter crescentus* has the propensity to attach to surfaces as part of its transition into the stalked S-phase of its life cycle. This behavior is heavily influenced by intracellular c-di-GMP and thus poses H-NOX as a potential influencer of *C. crescentus* surface attachment and cell cycle. By generating a strain of *C. crescentus* lacking *hnox*, our laboratory has demonstrated that this strain exhibits a considerable growth deficit, an increase in biofilm formation, and an elevation in c-di-GMP. Furthermore, in our comprehensive proteome study of 2779 proteins, 236 proteins were identified that exhibited differential expression in *Δhnox C. crescentus*, with 132 being downregulated and 104 being upregulated, as determined by a fold change of ≥1.5 or ≤0.66 and a *p* value ≤0.05. Our systematic analysis unveiled several regulated candidates including GcrA, PopA, RsaA, FtsL, DipM, FlgC, and CpaE that are associated with the regulation of the cellular division process, surface proteins, flagellum, and pili assembly. Further examination of Gene Ontology and pathways indicated that the key differences could be attributed to several metabolic processes. Taken together, our data indicate a role for the HNOX protein in *C. crescentus* cell cycle progression.

## Highlights

• H-NOX is a sensor protein regulating biofilm formation in bacterial species
• Deletion of *hnox* in *Caulobacter crescentus* disrupts cell cycle progression
• A comprehensive proteome *Caulobacter crescentus* is presented
• H-NOX regulates cell cycle, pili, flagella, biofilm, and surface layer proteins
• H-NOX proteins are not previously known to participate in cell cycle regulation

## Graphical Abstract

**Figure:**
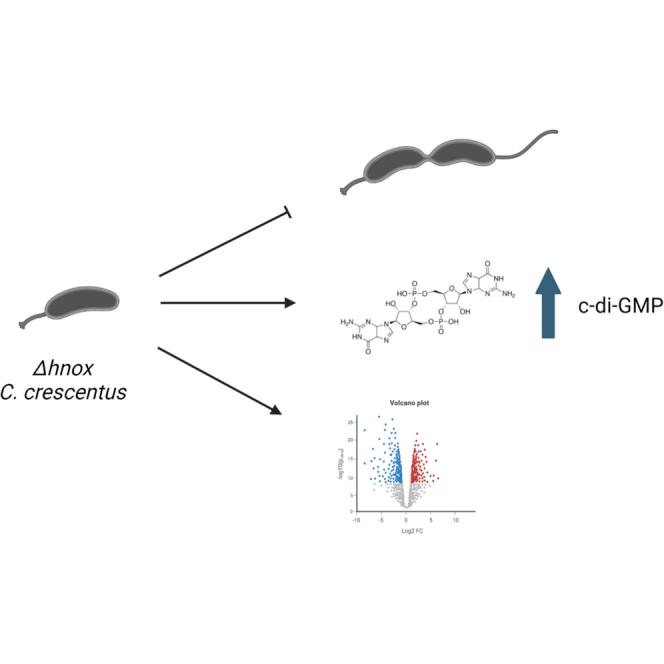

## In Brief

The heme nitric oxide/oxygen binding protein (H-NOX) in bacteria is a diatomic gas sensor that utilizes heme to signal for responses to environmental stimuli. Upon deletion of the *hnox* gene in *Caulobacter crescentus*, there is a notable growth deficiency, increase in intracellular c-di-GMP and differential expression of several proteins including some pertaining to differentiation and cell cycle progression (Created with BioRender.com).

*Caulobacter crescentus*, also known as *Caulobacter vibrioides*, is a prototypical organism for studying bacterial cell cycle and replication mechanisms. This is due largely to its dimorphic life cycle characterized by two discrete morphological forms, the swarmer and the stalked cell. The swarmer cell is a motile nonreplicative form with a solitary polar flagellum and multiple type IV pili localized to the flagellar pole. The transition to the replicative sessile stalked cell is characterized by the loss of these structures and the growth of a cell wall extension called the stalk, which adheres the cell to various surfaces through an adhesive holdfast structure at its tip. Only upon transition to a stalked cell will *C. crescentus* initiate genome replication and subsequently proceed to cellular division to produce a daughter swarmer cell (1). These dynamic cellular processes are controlled by multiple cell cycle regulators that have been thoroughly investigated with regard to their temporal expression, localization, and interactions (2, 3, 4).

The *C. crescentus* holdfast is essential to adhere to surfaces and the subsequent formation of communities of cells commonly known as a biofilm (5, 6, 7). Biofilms often confer resistance to antimicrobial treatments as well as protection from changes to the local environment. As such, they have been recognized as a potential threat to industrial operations because of biofouling or the medical sector because of nosocomial infections, recurring infections, and antibiotic resistance within pathogenic species (6, 8, 9). The life cycle of *C. crescentus* is intimately connected with surface attachment, making it an interesting model system for the study of biofilm formation and dynamics.

A major determinant in the transition from sessile to motile lifestyles in bacteria is the secondary messenger cyclic diguanylate monophosphate (c-di-GMP). C-di-GMP interacts with various effector proteins, transcription factors, and other entities to regulate virulence factors, cell cycle progression, and biofilm formation (10). Intracellular c-di-GMP levels are determined by the activities of diguanylate cyclase (DGC) enzymes that synthesize it from GTP and phosphodiesterases (PDEs) that break it down. These in turn are tightly regulated by various means. In *C. crescentus*, increased c-di-GMP production by DGC enzymes is a critical determinant in the G1/S transition and transformation to stalked cells (11, 12, 13, 14). However, the means by which the DGC enzymes are regulated is not entirely clear.

A family of heme-based sensor proteins called heme nitric oxide/oxygen binding protein (H-NOX) has been linked to c-di-GMP regulation and biofilm formation in a number of bacterial species (15). The H-NOX proteins from facultative anaerobes selectively bind nitric oxide (NO), which alters their interaction with downstream effector proteins, typically DGC/PDE enzymes or histidine kinases (HKs) (16). Thus, H-NOX can act as a primary regulator of c-di-GMP metabolism, either regulating DGC/PDE activity directly or indirectly through phosphorylation by the cognate HK. The *C. crescentus* genome encodes an H-NOX protein (CC2992/CCNA_03087) adjacent to an HK, HnoK (CC2993/CCNA_03088), suggesting that it may control a phosphorylation network influencing c-di-GMP metabolism, cell cycle, and development in *C. crescentus* (15, 17, 18).

Here, we assess the function of *C. crescentus* H-NOX using an *hnox* deletion strain. The results indicate significant perturbation in cell growth and cell cycle progression in the deletion strain as evidenced by slow growth, a decreased proportion of swarmer cells, and increased intracellular c-di-GMP. In addition, we note an increase in osmotic stress in this strain indicating a yet unidentified role for H-NOX. This was followed up by a quantitative proteomics analysis, which showed H-NOX-dependent protein alterations in a total of 236 proteins including key cell cycle regulators, surface proteins, and proteins involved in flagellar biogenesis and pili formation. To our knowledge, this is the most comprehensive proteomics analysis of *C. crescentus* provided to date and the first time H-NOX has been implicated in cell growth and division.

## Experimental Procedures

### Experimental Design and Statistical Rationale

Proteome analyses were conducted on WT and Δ*hnox C. crescentus* strains cultured in three biological replicates. These replicates were processed together, and the proteins were digested using trypsin as described later. Peptides were labeled with the tandem mass tag (TMT) 10-plex isobaric label reagent set (Thermo) and then combined into a single multiplex sample group. The proteome samples were analyzed using LC–MS/MS. All peptides and proteins that were uniquely identified, both quantified and nonquantified, were reported in supplemental Table S1. Proteins with significantly differential abundance between strains were identified by applying a two-sided *t* test with a cutoff of log_2_ fold change greater than 0.58 and a *p* value less than 0.05. These are reported in supplemental Table S2. The *sample preparation and data analysis* sections provide a comprehensive discussion of the various sample preparation optimization experiments, growth conditions, mass spectrometry (MS) parameters, and analysis parameters.

### Strains and Media

*Escherichia coli* DH5α cells were grown in LB media and on LB agar (1.5% agar) plates containing 25 μg/ml kanamycin at 37 °C. WT and Δ*hnox C. crescentus* strains were grown in peptone yeast extract (PYE) (0.2% [w/v] peptone, 0.1% [w/v] yeast extract, 1 mM MgSO_4_, and 0.5 mM CaCl_2_) media or in M2 minimal media containing 0.2% glucose (M2G) (1× M2 salts [17.4 g/l Na_2_HPO_4_, 10.6 g/l KH_2_PO_4_, 10 g/l NH_4_Cl], 0.5 mM MgSO_4_, 0.2% glucose, 0.1% ferrous sulfate chelate solution, and 0.5 mM CaCl_2_) supplemented with 100 μg/ml ampicillin. *C. crescentus* liquid cultures were grown with consistent shaking at 28 °C.

### Generation of Δ*hnox* Strain of *C. crescentus*

The *hnox* deletion strain was generated as described previously (19). Briefly, homology regions flanking *hnox* were amplified by PCR and cloned into the suicide plasmid pNPTS138 at the BamHI restriction site using the Gibson cloning method (20). The resulting plasmid was introduced to ATCC19089 *C. crescentus* cells by electroporation. Double recombinants were initially selected by kanamycin resistance followed by counterselection against sucrose. All plasmids and the *hnox* deletion strain were verified by DNA sequencing. The upstream primers used were FWD: 5′-ggccgaagctagcgaattcgtggacataggcggcgcaatcagc-3′ and REV: 5′-gaaaacgggatgacggcgctagtcatggcgccgcccgtcc-3′. The downstream primers used were FWD: 5′-gaacgggacgggcggcgccatgactagcgccgtcatccc-3′ and REV: 5′-gcttctctgcaggatatctgctggtcgagaaaggcgagcgt-3′.

### Growth, Viability, and Osmotic Stress Tolerance

Overnight cultures of *C. crescentus* WT and Δ*hnox* strains were grown in PYE with or without supplementation with ampicillin at 100 μg/ml. These were used to inoculate 96-well microplates to a final volume of 100 μl and absorbance of 0.05 at 600 nm. Cell growth at 30 °C was monitored with hourly measurements for 24 h with orbital shaking at 225 RPM in BioTek EPOCH 2 plate reader.

For viability determinations, *C. crescentus* was grown in PYE supplemented with ampicillin at 100 μg/ml to an absorbance of 0.5 at 600 nm. Cultures were then serially diluted to 10^−7^ and plated on PYE supplemented with ampicillin using 5 μl of each dilution. To determine the effect of osmotic stress on cells, this same process was repeated using PYE plates containing 100 μg/ml ampicillin and 50 mM KCl. Plates were imaged, and colonies were counted to demonstrate differences in viability.

### Biofilm Measurement

Surface attached biomass was measured by crystal violet staining after 24 h of growth. Well plates were rinsed three times with deionized water and then stained with 150 μl of a 0.1% w/v crystal violet solution. Plates were shaken for 15 min following crystal violet addition and were rinsed three times with deionized water again before the stain was lifted by the addition of 200 μl of dimethyl sulfoxide. The ratio of absorbance at 570 nm (crystal violet-stained biomass) to absorbance at 600 nm (absorbance of cells at the time they were removed from the plate reader) was used to provide an estimate of relative biofilm formation.

### Stalked Population Estimates

Liquid cultures of WT and Δ*hnox C. crescentus* were grown in PYE supplemented with ampicillin to an absorbance of 0.5 at 600 nm. About 1 μl of this culture was then added to a 2% PYE agar pad on a glass microscope slide and covered with a glass coverslip. Cells were visualized using an AxioObserver inverted microscope set to the phase contrast setting. A minimum of eight images were used for each strain (8 WT and 10 Δ*hnox*). The focal plane with the most clearly visible stalks was used for analysis. All cells that could be clearly distinguished were counted, and stalked cells were identified as described previously (21). A total of 601 WT cells and 1243 Δ*hnox* cells were counted. A ratio of stalked cells to the entire population was calculated for both WT and Δ*hnox* strains.

### Cell Length Analyses

Liquid cultures of WT and Δ*hnox C. crescentus* were grown in PYE supplemented with ampicillin to an absorbance of 0.5 at 600 nm. About 1 μl of this culture was then added to a 2% PYE agar pad on a glass microscope slide and covered with a glass coverslip. Cells were visualized using an AxioObserver inverted microscope set to the phase contrast setting. *C. crescentus* WT and Δ*hnox* cell lengths were determined using MicrobeJ (22) and identified upon having a minimum length and width of 0.5 μm.

### Synchronization

*C. crescentus* WT and Δ*hnox* strains were synchronized *via* centrifugation as described elsewhere (23). Cells were grown in a 5 ml overnight culture of PYE at 28 °C. These cultures were then diluted into 15 ml of M2G minimal media. Cells were grown to midlog phase and pelleted at 6400*g* for 10 min. Supernatant was removed, and the pelleted cells were washed with 1 ml of cold M2 media in a microcentrifuge tube. The washed cell pellet was resuspended in 800 μl of M2 and placed on ice. A 40% colloidal silica solution was diluted to 20% with M2 media, and 800 μl of the resulting solution was added to the resuspended cell pellet. The resulting mixture was centrifuged at 20,000*g* for 20 min. After centrifugation, two distinct layers were formed, the top layer containing predivisional and stalked cells and the bottom layer of swarmer cells. The top layer was aspirated off, and the swarmer layer was collected for future use after being pelleted and washed twice to remove silica.

A similar protocol was used for large cultures for c-di-GMP quantitation (23). The 15 ml M2G cultures were transferred to 1 l M2G and grown to a midexponential phase. Cells were then pelleted and resuspended with 180 ml M2 media. After the cells were resuspended, 60 ml of 40% colloidal silica was added to the mixture. The mixture was evenly distributed between eight centrifuge tubes and centrifuged at 20,000*g* for 30 min. The predivisional/stalked band was removed, and the swarmer band was collected, pelleted, and washed twice to remove silica, and stored in 15% glycerol at −80 °C for future use. Swarmer cell presence was verified for both large- and small-scale synchronies by phase contrast microscopy.

### C-di-GMP Quantitation

Synchronized *C. crescentus* was added to 100 ml PYE media supplemented with ampicillin to an absorbance of 0.05 at 600 nm. These cells were then incubated at 28 °C with shaking at 165 rpm until they reached an absorbance of 0.5 at 600 nm. Cells were then pelleted at 4000*g*, and c-di-GMP was extracted using an adapted protocol from Roy *et al*. (24). The pellet was washed twice with 1 ml of ice-cold 1× PBS and then resuspended in 100 μl of 1× PBS and boiled at 100 °C for 5 min. About 95% ethanol was then added to a final concentration of 65%, and the solution was vortexed for 15 s. The solution was then centrifuged at 16,500*g* for 2 min, and the supernatant was collected. This process was repeated twice more, and the combined supernatant was dried *via* speed vac.

Samples were analyzed using a Shimadzu triple quadrupole mass spectrometer (LCMS-8050). A SeQuant ZIC-HILIC column (3.5 μm, 200 Å, 100 × 2.1 mm) was used to separate the samples. Mobile phases consisted of 10 mM ammonium acetate in water (A) and 10 mM ammonium acetate in acetonitrile and water (95:5) (B). The gradient started at 5% A for 3 min, ramped from 5% to 40% A over 7 min, and held at 40% A for a wash step, followed by re-equilibration with 5% A for 7 min. The total run time was 20 min, and the flow rate was 0.5 ml/min. The column oven was set to 40 °C, and the sample injection volume was 5 μl.

The samples were analyzed by multiple reaction monitoring (MRM) in positive electrospray ionization mode. MS conditions were as follows: the nebulizer gas flow rate was 2 l/min, the drying gas flow rate gas was 10 l/min, the heating gas flow rate was 2 l/min, the desolvation temperature was 300 °C, and the electrospray voltage was 4 kV. Nitrogen was used as both nebulizing and drying gas, and argon was used as the collision gas. The product ions for cyclic di-GMP (*m/z* 691) were detected using three MRM transitions: *m/z* 152, 540, and 248 (25).

### Proteome Sample Preparation

Synchronized *C. crescentus* was added to 10 ml PYE media supplemented with ampicillin and incubated at 28 °C with shaking at 165 rpm until they reached an absorbance of 0.5 at 600 nm. Cells were then pelleted at 4000*g*, washed twice with ice-cold PBS, and cell pellets were stored at −80 °C prior to analysis. Cell pellets were resuspended in radioimmunoprecipitation assay buffer (Pierce) containing protease and phosphatase inhibitors (1:100 dilution), sonicated for 30 s, and centrifuged at 14,000*g*. About 100 μl of extracted total protein at 1 mg/ml was reduced by incubation with 10 mM Tris(2-carboxyethyl) phosphine hydrochloride at 37 °C for 30 min followed by alkylation with 30 mM iodoacetamide at 37 °C for 60 min in the dark. Chloroform–methanol extraction was then performed by adding 30 μl of 10× PBS followed by 400 μl of methanol, 100 μl of chloroform, and 270 μl of 1× PBS, vortexing after each step. The sample was centrifuged at 14,000*g*, and the aqueous layer was removed and discarded. Another 400 μl of methanol was added, vortexed, and centrifuged at 20,000*g* for 5 min, and the supernatant was removed. This step was repeated once, and the sample was allowed to air dry. The protein pellet was resuspended in 100 mM triethylammonium bicarbonate, and sequencing grade trypsin (Promega) was added to a ratio of 1:50 by mass. Samples were incubated for 18 h at 37 °C and desalted using Sep-Pak C18 plates (Waters).

Purified peptides were labeled using a TMT 10-plex isobaric label reagent set (Thermo) and then consolidated into a singular multiplex sample group. The labeled peptides were separated into 46 fractions through the employment of a 100 × 1.0 mm Acquity BEH C18 column (Waters) with the aid of an UltiMate 3000 UHPLC system (Thermo). This process was carried out using a 50 min gradient that ranged from 99:1 to 60:40 buffer A:B ratio under basic pH conditions. The resulting fractions were then consolidated into 18 superfractions. Subsequently, each superfraction underwent additional separation *via* reverse-phase XSelect CSH C18 2.5 μm resin (Waters) utilizing an in-line 150 × 0.075 mm column on an UltiMate 3000 RSLCnano system (Thermo). The peptide elution was carried out by implementing a gradient over a period of 75 min, transitioning from a buffer A:B ratio of 98:2 to 60:40. The peptides that were eluted experienced ionization through electrospray at a voltage of 2.4 kV. Subsequently, these peptides were subjected to mass spectrometric analysis using multinotch MS3 parameters on an Orbitrap Eclipse Tribrid mass spectrometer manufactured by Thermo. The MS data were obtained utilizing the Fourier transform MS analyzer in the high-speed profile mode with a resolution of 120,000 across a spectrum range of 375 to 1500 *m/z*. After collision-induced dissociation activation with a normalized collision energy of 31.0, MS/MS data were obtained using the ion trap analyzer in centroid mode and normal mass range. The study employed synchronous precursor selection to choose a maximum of 10 MS/MS precursors for higher-energy collisional dissociation activation, with a normalized collision energy of 55.0. Subsequently, MS3 reporter ion data were acquired using the Fourier transform MS analyzer in profile mode, with a resolution of 50,000 over a range of 100 to 500 *m/z*.

### Data Analysis

To study the impact of *hnox* deletion on the global proteome of *C. crescentus*, triplicate cultures of synchronized cells were grown to exponential phase, collected, and extracted proteins were labeled with TMTs and analyzed by LC–MS/MS. The protein identification and quantification of MS3 reporter ions was carried out through the utilization of MaxQuant (version 2.2.0.0, Max Planck Institute of Biochemistry) software (26). The UniPProtKB *C. vibrioides* (UP000001364, October 2022) database was employed for this purpose, with a parent ion tolerance of 3 ppm, a fragment ion tolerance of 0.5 Da, and a reporter ion tolerance of 0.003 Da. For variable modifications, we selected oxidation of methionine and acetylation of the protein N terminus. In addition, we set carbamidomethylation of cysteines as the fixed modification. Through searching the database, 2779 protein entries were identified. The Scaffold Q + S software, developed by Proteome Software, was employed to validate peptide and protein identifications based on MS/MS analysis. For a protein identification to be considered valid, it had to meet two criteria: (1) the false discovery rate to be set to 1% on the peptide-spectrum match and protein level using the implemented decoy algorithm and (2) at least one peptide had to be identified. The Protein Prophet algorithm was used to assign protein probabilities as described previously (27). In addition, the software was used to conduct statistical analysis based on reporter ions. The assessment of the quality of TMT MS3 reporter ion intensity values for protein and their normalization is conducted using ProteiNorm (ByrumLab) (28). The data were normalized using the variance stabilization normalization method as proposed by Huber *et al*. (29). Subsequently, the Linear Models for Microarray Data (limma) approach was utilized for statistical analysis. The application of Empirical Bayes smoothing to the standard errors was carried out in accordance with the methodology outlined by Ritchie *et al*. (30). The data analysis and visualization were conducted using Python. The significant proteins were identified by adjusting a two-sided *t* test with a cutoff of log2 fold change greater than 0.58 and a *p* value less than 0.05.

### RT–Quantitative PCR

Synchronized WT and mutant cells were grown in PYE supplemented with ampicillin to an absorbance of 0.5 at 600 nm. About 4 ml of 5% v/v phenol in ethanol was added to 10 ml cells, chilled on ice for 30 min, centrifuged, and the pellet was stored at −80 °C. RNA was extracted using a PureLink RNA Mini Kit (Invitrogen). DNA contamination was removed using an on-column DNase digestion protocol (Invitrogen). RNA concentration and purity were determined spectrophotometrically using a NanoDrop 2000 Spectrophotometer (Thermo). Complementary DNA was synthesized from 2 μg of pure RNA in a 20 μl reaction volume using the SuperScript III first-strand synthesis kit (Invitrogen). Complementary DNA was diluted with nuclease-free water to a final concentration 10 ng/μl and used for real-time PCRs. The primers were designed to amplify 100 to 150 base pairs (bp) of target genes with an average *T*_m_ ∼55 °C and were used at a final concentration of 0.3 μM. Quantification of amplified PCR product using PowerTrack SYBR Green PCR Master Mix (Applied Biosystems) was monitored by CFX-Connect real-time system (Bio-Rad). The relative expression of genes was normalized to *rpoH*, a housekeeping gene encoding a sigma factor previously used for real-time PCR experiments in this organism (31).

## Results

### Δ*hnox C. crescentus* Exhibits Inhibited Cell Growth and a Relative Increase in Surface-Attached Biomass

**Fig. 1:**
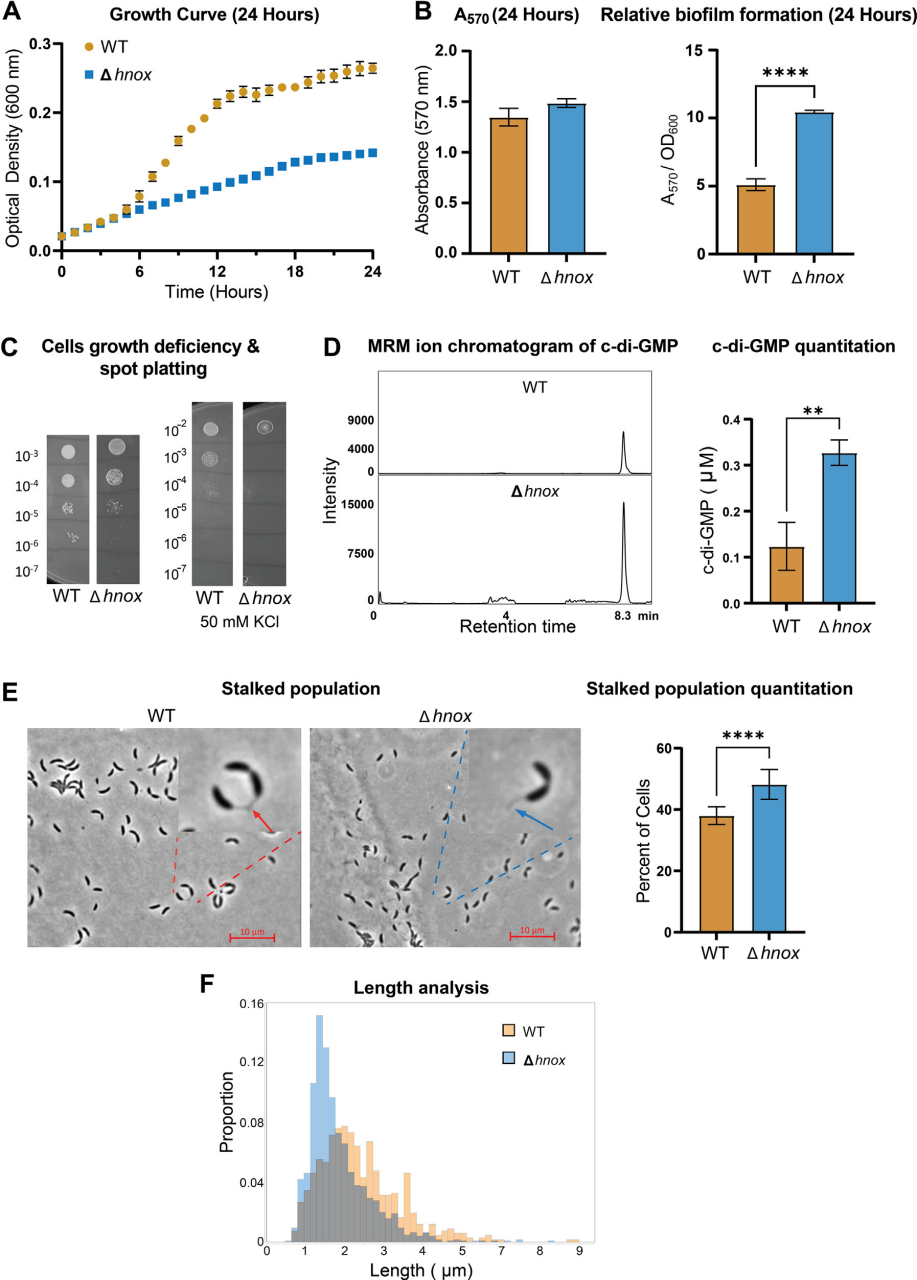
**Impact of *hnox* deletion in *Caulobacter crescentus* cell growth, surface attachment, and cell type population.***A*, the growth curves of WT and Δ*hnox* strains of *C. crescentus*. Readings of absorbance at 600 nm were taken every hour for 24 h, and each strain was measured in quadruplicate. Error bars are representative of one standard deviation above and below the mean for each measurement. *B*, measurement of crystal violet staining of the well plate in (*A*) using absorbance at 570 nm after the stain was dissolved in DMSO (*left*). The relative biofilm formation is expressed as a ratio of absorbance at 570 nm/absorbance at 600 nm (*right*). *C*, serial dilutions of *C. crescentus* grown to absorbance of 0.5 at 600 nm spot-plated on PYE + ampicillin or PYE + ampicillin + 50 mM KCl. Dilutions of the original cultures are represented on each side of the image. *D*, representative multiple reaction monitoring (MRM) chromatogram of c-di-GMP collected using LC–MS/MS (*left*) and c-di-GMP quantitation (*right*). *E*, WT and Δ*hnox C. crescentus* phase contrast images (*left*). Populations of stalked cells calculated in reference to total cell count (*right*). Percentages are based on 601 counted WT cells and 1243 counted Δ*hnox* cells. *F*, cell length distributions as determined by MicrobeJ. The median cell lengths are 2.156 and 1.601 μm for WT and Δ*hnox C. crescentus,* respectively. (∗*p* ≤ 0.05, ∗∗*p* ≤ 0.01, ∗∗∗*p* ≤ 0.001, and ∗∗∗∗*p* ≤ 0.0001). c-di-GMP, cyclic diguanylate monophosphate; DMSO, dimethyl sulfoxide; PYE, peptone yeast extract.

WT and Δ*hnox C. crescentus* growth were monitored by absorbance at 600 nm in a 96-well plate for 24 h. After a few hours of growth, a significant growth defect was noted in Δ*hnox versus* WT (Fig. 1*A*). The well plates were stained with crystal violet, and the *Δhnox* strain demonstrated a relative increase in surface-attached biomass as indicated by the ratio of staining to absorbance (Fig. 1*B*). A hyperbiofilm phenotype was also noted for the *hnox* deletion in *Legionella pneumophila* and the *hnox/nosp* double knockout of *Shewanella oneidensis* (32, 33). To our knowledge, there has been no identified effect on growth rate when knocking out *hnox* in other species.

### Δ*hnox C. crescentus* Demonstrates Decreased Cell Viability and Increased Sensitivity to Osmotic Stress

The viability of the Δ*hnox* strain was assessed by comparing colony-forming units to the WT organism. The WT and Δ*hnox* strains were spot plated on PYE plates supplemented with ampicillin at midexponential phase. Cultures were grown to an absorbance of 0.5 at 600 nm, and serial dilutions were plated to assess viability. Plates were then incubated for 48 h at 28 °C. A visible decrease in colonies for the mutant strains suggests that cell viability was negatively impacted by the mutation (Fig. 1*C*). Cells were also plated on PYE plates supplemented with ampicillin and 50 mM KCl to assess sensitivity to osmotic stress. Under these conditions, the mutant strain exhibits a dramatic decrease in viability relative to WT.

### C-di-GMP Production is Increased in the Δ*hnox* Strain of *C. crescentus*

C-di-GMP has been linked to biofilm formation across several bacterial species (34), and H-NOX homologs have been associated with the regulation of c-di-GMP (16, 32). To investigate the potential involvement of H-NOX in the regulation of c-di-GMP in *C. crescentus*, a tandem LC–MS/MS approach was utilized. By adapting a method by Roy *et al*. (24) c-di-GMP was extracted from *C. crescentus* cells grown to midexponential phase and quantified using MRM. The Δ*hnox* strain of *C. crescentus* showed an elevated intracellular c-di-GMP pool relative to WT (Fig. 1*D*). This is consistent with increased surface-attached biomass, as stalked *C. crescentus* cells typically exhibit larger concentrations of c-di-GMP than their swarmer counterparts (35). It has been noted that as swarmer cells age, intracellular concentrations of c-di-GMP will increase until they reach a stalked phase (11, 36).

### Stalked Cells are More Abundant in Δ*hnox C. crescentus*

Our observed changes to c-di-GMP upon knocking out *hnox* imply a higher ratio of stalked or late-stage swarmer cells in the Δ*hnox* cultures than in their WT counterparts. To confirm this, we used phase contrast microscopy to differentiate stalked and swarmer cells. After applying a pre-established methodology for cell counting (21), it was observed that the relative population of stalked cells in Δ*hnox* is significantly higher relative to WT (Fig. 1*E*). These data are consistent with our previous observation of increased surface-attached biomass and decrease in cell growth and implies a disruption to the *C. crescentus* cell cycle. In addition, microscopy images analyzed using MicrobeJ (22) indicate an apparent difference in median cell length between the WT and Δ*hnox* strain of *C. crescentus* (Fig. 1*F*). This could in part be related to an underlying disruption to cell envelope homeostasis and stress responses (37, 38).

### TMT-Based Proteome Analysis of *Δhnox C. crescentus*

**Fig. 2:**
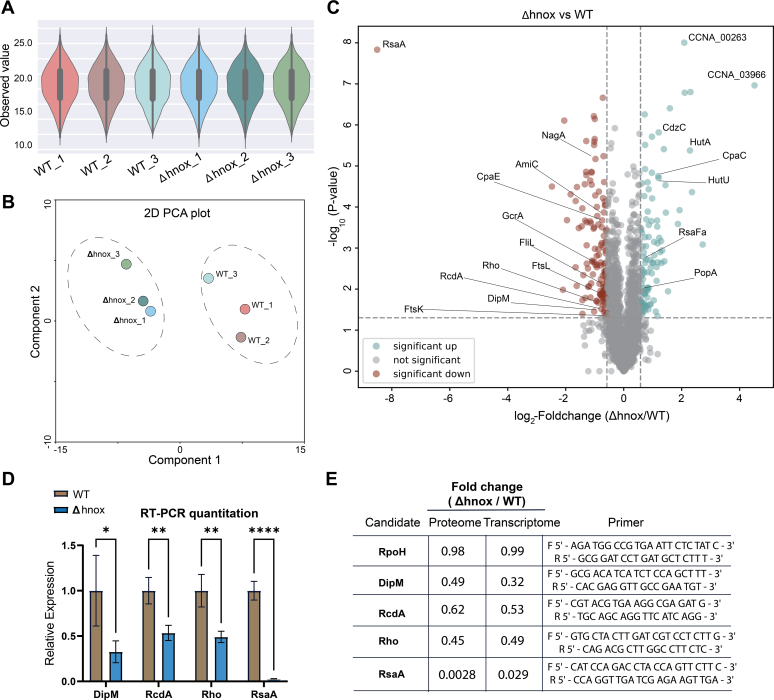
**Proteome analysis of *Caulobacter crescentus***. *A*, the violin plots illustrate comparable protein intensity distributions between the Δ*hnox* and WT *C. crescentus. B*, principal component analysis (PCA) of six *C. crescentus* samples. The results obtained from PCA indicate a distinct separation between the WT and Δ*hnox* samples. *C*, a volcano plot displays the quantification of protein classes that are consistent across three replicates of each strain. Significant upregulated and downregulated proteins in Δ*hnox* are highlighted in *blue* and *red*, respectively. *D*, real-time quantitative PCR measurements of selected candidates in WT (*gold*) and Δ*hnox* (*blue*) *C. crescentus*. Relative expression level values are an average of three technical replicates and four biological replicates for each candidate. Error bars indicate mean ± SEM. (∗*p* ≤ 0.05, ∗∗*p* ≤ 0.01, ∗∗∗*p* ≤ 0.001, and ∗∗∗∗*p* ≤ 0.0001). *E*, comparative analysis of the expression of selected candidates (fold change ≥1.5, *p* value ≤0.5) at the proteomic and transcriptomic levels. RpoH was used to normalize the data. The last column contains primer sequences used. (∗*p* ≤ 0.05, ∗∗*p* ≤ 0.01, ∗∗∗*p* ≤ 0.001, and ∗∗∗∗*p* ≤ 0.0001).

To further characterize the function of H-NOX and the signaling pathway in which it participates, we employed a TMT proteomics approach (39) to rigorously quantify protein abundances in WT and Δhnox samples. With the current methodology, we were able to profile the Δ*hnox* and WT *C. crescentu*s strains in triplicate in less than 12 h of measuring time, with an average rate of 18.6 protein quantifications per minute for each sample. In total, 2779 proteins were identified, along with 27,782 unique peptide sequences connected to 97,794 MS/MS spectra (supplemental Table S1). An average of 2770 proteins were identified in each sample and were highly reproducible across multiple LC–MS/MS runs with comparable setups (supplemental Fig. S1). Subsequently, an analysis was conducted on the protein intensity distribution. The results, as depicted in Figure 2*A*, through a violin plot, indicated a consistent pattern across all the samples. The multiplot scattering plot exhibits the Pearson's correlation coefficients among biological triplicates and showed a remarkably high level of correlation (*r* > 0.9) (supplemental Fig. S2). Next, we conducted principal component analysis for both Δ*hnox* and WT samples to examine the global consequences of the hnox deletion in *C. crescentus*. The principal component analysis accounted for approximately 80% of the variance between the first two components (data not shown). We observed a distinct segregation between the WT and Δhnox samples (Fig. 2*B*), illustrating that H-NOX status has a perceptible impact on the total proteome. Next, we assessed the shared and unique protein alterations of the Δ*hnox* samples when compared with the WT samples. In the present analysis, a hierarchical clustering was conducted to show the biological link between certain genes in Δ*hnox* and WT *C. crescentus*. Notably, the findings showed that most of the Δ*hnox* and WT samples could be easily distinguished from one another (supplemental Fig. S3).

Differential expression analysis identified 234 proteins with altered abundance in Δ*hnox* compared with WT samples (fold change ≥1.5, *p* value ≤0.05) as shown in volcano plot (Fig. 2*C*) (supplemental Table S2). Proteome data were further validated using RT–quantitative PCR to confirm changes in relative expression for several identified candidates (Fig. 2, *D* and *E*). The most dramatic change was for the surface layer protein RsaA, which was downregulated approximately 350-fold and 35-fold in protein and transcript abundance, respectively.

**Fig. 3:**
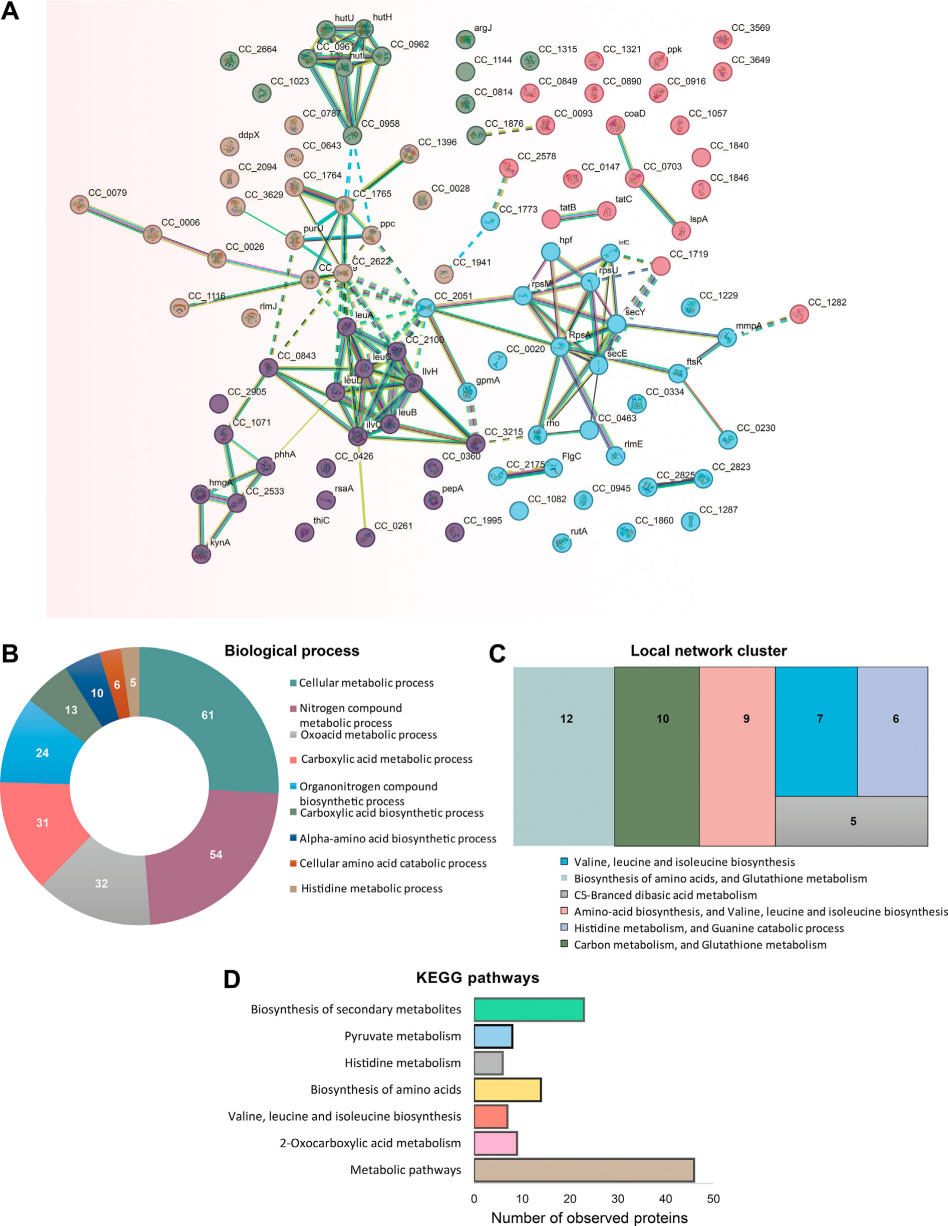
**Interactome study and Gene Ontology (GO) analysis of Δ*hnox* and WT *Caulobacter crescentus***. *A*, K-means clustering within the framework of interaction networks. There are five separate groups into which 102 interactive candidates have been placed. *B* and *C*, GO study of Δ*hnox* in *C. crescentus*. The pie chart reveals the biological process of 54 proteins engaged in nitrogen compound metabolism, whereas neighbor networks indicate 15 proteins involved in amino acid biosynthesis and glutathione metabolism. *D*, the Kyoto Encyclopedia of Genes and Genomes (KEGG) pathways demonstrate the association of regulated proteins in various pathways, including but not limited to the biosynthesis of secondary metabolites, biosynthesis of amino acids, and metabolic pathways.

To investigate the potential intermolecular connections among differentially expressed proteins (supplemental Table S2) in WT and Δ*hnox* strains, an interaction analysis was carried out utilizing the Search Tool for Retrieval of Interacting Genes (STRING) (40). The implementation of K-means clustering within protein–protein interaction networks placed 102 of our 236 differentially expressed proteins into five discrete clusters differentiated by subcellular compartments or biological processes including outer membrane, ribosomal component, and branched-chain amino acid biosynthesis (Fig. 3*A*). A subsequent Gene Ontology analysis identified 32 proteins that correspond with oxoacid metabolism and 54 proteins that participate in the metabolism of nitrogen compounds (Fig. 3*B*). The local network cluster analysis (Fig. 3*C*) and Kyoto Encyclopedia of Genes and Genomes pathway analysis (Fig. 3*D*) confirmed a correlation between H-NOX status and oxoacid metabolism, as well amino acid metabolism, particularly histidine and C5-branched dibasic acid metabolism. Furthermore, disrupted glutathione metabolism was implicated with four predicted glutathione-*S*-transferase proteins (CCNA_02936, CCNA_00818, CCNA_01602, and CCNA_01376) and one lactoglutathione lyase (CCNA_03193) significantly upregulated in the Δ*hnox* strain.

Thus, the comprehensive proteome data presented herein suggest a potential role for the H-NOX protein in regulating several metabolic processes. Furthermore, manual inspection identified several hits involved in cell cycle regulation, cell division, flagellum and pili assembly, and cell envelope maintenance (Table 1). Each category and its relevance to the observed Δ*hnox* phenotype are discussed in detail later.

**Table 1:** Differential abundance of proteins involved in various cell processes

Function	UniProt ID	Gene name	Description	Log_2_ fold change (Δhnox/WT)	*p* (−Log_10_)
Cell envelope maintenance	A0A0H3C8J1	*rsaA*	S-layer protein RsaA	−8.51	7.83
	A0A0H3C8J5	*perA*	Perosamine synthetase PerA	0.65	2.47
	A0A0H3C731	*rsaFa*	Type I secretion outer membrane protein RsaFa	0.70	2.78
	A0A0H3C437	*chvI*	Two-component response regulator ChvI	1.16	3.52
	A0A0H3CDX1	*rfbB*	dTDP-glucose 4,6-dehydratase	1.01	2.64
Cell cycle regulation	A0A0H3CD07	*rcdA*	Regulator of CtrA degradation RcdA	−0.68	1.44
	A0A0H3C8I4	*popA*	Cyclic di-GMP effector protein PopA	0.70	1.90
	A0A0H3C9J4	*gcrA*	Cell cycle regulatory protein GcrA	−0.79	2.57
Cell division	A0A0H3C9I3	*amiC*	H-acetylmuramoyl-*L*-alanine amidase	−0.65	4.04
	A0A0H3CE63	*ftsK*	DNA translocase FtsK	−0.60	1.30
	A0A0H3CB25	*ftsL*	Cell division protein FtsL	−0.65	2.05
	A0A0H3C9Q9	*dipM*	Cell division protein DipM	−1.02	1.45
Flagellar protein	B8GXB6	*fliL*	Flagellar FliL protein	−0.59	2.33
	A0A0H3C743	*flgC*	Flagellar basal-body rod protein FlgC	−0.62	1.78
Pili protein	A0A0H3CC36	*cpaC*	Outer membrane pilus secretion channel CpaC	1.19	4.75
	A0A0H3CAK9	*cpaE*	Pilus assembly ATPase CpaE	−0.65	4.13
	A0A0H3CB91	*cpaI*	CpaC-related secretion pathway protein CpaI	−0.59	1.62
	A0A0H3CAK5	CCNA_03033	Flp pilus assembly protein TadD	0.60	3.48

## Discussion

In the current study, we generated a Δ*hnox* strain of *C. crescentus* that is characterized by a growth defect in liquid media, a relative increase in surface-attached biomass and stalked cells, and an increase in intracellular c-di-GMP. Multiple studies on various bacterial species have shown a connection between H-NOX and the modulation of biofilm in response to environmental factors. However, this is the first study to our knowledge to establish a role for H-NOX in cell cycle regulation, resulting in diminished cell division to produce daughter swarmer cells in *C. crescentus*. The total proteome data provide additional evidence for this and further implicate cell cycle arrest during S-phase as indicated by the altered abundance of proteins involved in histidine and glutathione metabolism, cell cycle regulation and division, pili and flagellar assembly, and cell envelope maintenance as discussed below.

### Histidine and Glutathione Metabolism

Metabolic cues are responsible for regulating various stages of the cell division process in *C. crescentus* (41, 42). For example, the dysregulation of cell growth and cell division has been found to be associated with nitrogen metabolism (43). Furthermore, the regulation of oxidative stress in *C. crescentus* is significantly influenced by the pivotal role played by glutathione. Under conditions of oxidative stress, an increase in the expression of genes related to the production of histidine and proteins associated with glutathione metabolism has been observed (44).

### Cell Cycle Regulation and Cell Division

**Fig. 4:**
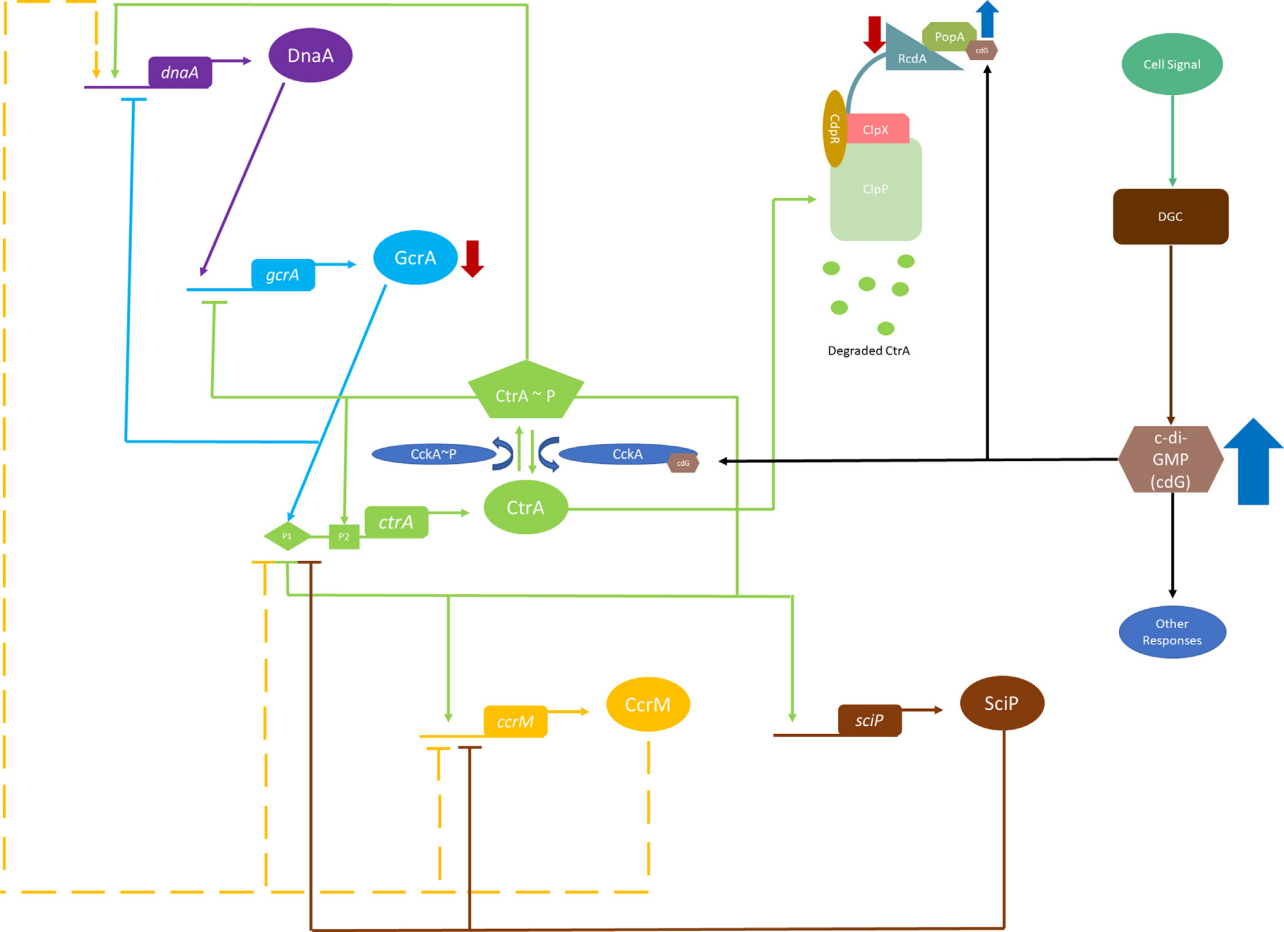
**Cell cycle regulatory networks of *Caulobacter crescentus***. With an *arrow* or *bar*, *solid lines* show how master regulators (DnaA, GcrA, CcrM, CtrA, and SciP) are activated or inhibited, respectively. The effects of CcrM methylation on the dnaA, ctrA, and ccrM genes are shown by the *dashed lines*.

*C. crescentus* has been a model organism to study bacterial cell cycle progression because of its ease of synchronization (45). In addition, temporal regulation of its cell cycle regulators has been well documented. *C. crescentus* has five “master” cell cycle regulators including DnaA, GcrA, CtrA, CcrM, and SciP (4, 46, 47, 48, 49) (Fig. 4). Throughout progression of the cell cycle, these proteins act to regulate each other as well as several other cell cycle–dependent proteins. The transcription factor DnaA facilitates the transcriptional activation of *gcrA*, which in turn promotes the activation of *ctrA* (50). Upon phosphorylation by the bifunctional kinase/phosphatase CckA, CtrA-P represses *gcrA* expression, stimulates *dnaA*, *ccrM*, and *sciP* expression (49, 50, 51), and blocks replication initiation by binding to the origin. Cellular levels of c-di-GMP rise in the G1 to S phase transition, due at least in part to the increased activity of the DGC enzyme PleD. Elevated c-di-GMP binds to CckA, switching its activity from kinase to phosphatase, dephosphorylating CtrA and allowing replication to proceed. A notable observation of our proteomics data was the downregulation of the master cell-cycle regulator GcrA in Δ*hnox*. GcrA exerts an influence on the expression of more than 200 genes that are associated with growth and cell cycle (52). Of these, we have identified differential expression of CpaC and CpaE. However, these genes are also regulated by CtrA (53, 54). Given that the regulation of GcrA is contingent on DnaA and CtrA, and neither has exhibited differential expression in our proteomics data, it is plausible that the downregulation of GcrA could be attributed primarily to changes in the phosphorylation state of CtrA.

Furthermore, we observed a decrease in the expression of RcdA and an increase in the expression of PopA. These proteins act as adapters to a CpdR-bound ClpXP protease in *C. crescentus* (55) (Fig. 4). Upon dephosphorylation by CckA, CdpR is activated and participates in interaction with ClpXP (56). Subsequently, the protease engages in interaction with RcdA for the purpose of breaking down targets, including TacA. Alternatively, RcdA may recruit c-di-GMP-bound PopA to degrade CtrA (55), and we observe an upregulation of PopA levels in Δ*hnox*. Taken together, alterations in the levels of c-di-GMP and regulator protein levels upon *hnox* deletion do not present a clear mechanism for cell cycle arrest in S-phase. Rather, they may point to compensatory mechanisms and alterations in post-translational modifications that ultimately result in the Δ*hnox* phenotype.

We also identified the expression profile of 13 cell division–related proteins, observing downregulation of three major candidates FtsK, FtsL, and DipM in the Δ*hnox* strain. FtsK plays a crucial role in the last phases of the cell cycle by facilitating cytokinesis and chromosomal segregation, aiding in the tight connection between these two processes in *C. crescentus* (57). DipM, a member of the LytM family, takes part in the coordinated constriction of the cell envelope layers at the division plane and the remodeling of the cell wall (58). Of note, lack of DipM results in delayed invagination of the cell wall and outer membrane during cytokinesis, which leads to severe division and polarity abnormalities. in *C. crescentus* (59). Overall, we observed a tendency of cell division proteins being downregulated in the Δ*hnox* group. Thus, the regulated expression of key participants in the cell cycle's final phases points to disturbed cell division machinery in the Δ*hnox* strain.

### Pili and Flagellar Assembly

By studying differential expression of proteins pertaining to polar appendages such as flagella and pili, we have been able to identify key elements that hint toward cell cycle disruption occurring in S-phase. The pili related proteins, CpaC, CpaE, CpaI, and TadD (also referred to as CpaO), are all differentially expressed, as are the flagella-related proteins, including FliL, FlgC, and PopA, which recruits other proteins to regulate flagellar assembly (60). Downregulation of CpaE in the Δ*hnox* mutant is consistent with prior findings that the transcription of CpaE is reduced during the majority of S-phase but increases during late S/G2-phase to establish the swarmer pole (61). Concurrently, an increase in the expression of CpaC is observed, a protein that is not typically present in the WT strain of *C. crescentus* until the transition from G1 to S-phase and reaches its highest level during the late S-phase (61). CpaI and TadD demonstrate downregulation and upregulation, respectively. It is hypothesized that CpaI functions as a CpaC secretin, and therefore, a plausible compensatory mechanism in reaction to CpaC overexpression could be downregulation of CpaI (54). TadD is described as a pilotin, indicating that its upregulation may be associated with the overexpression of CpaC.

The presence of FliL has been detected throughout all phases of the cellular division process in *C. crescentus*. Despite its absence of polar localization, FliL is essential for the appropriate operation of flagella. Therefore, the increased surface adhesion observed in the Δ*hnox C. crescentus* strain may be linked to the suppression of the flagellar protein, FliL (62). FlgC is a basal-body rod protein for the *C. crescentus* flagellum (63). While FliL is expressed throughout the cell cycle, downregulation of FlgC and a downward trend of other flagellar proteins are consistent with a higher stalked population in the Δ*hnox* strain of *C. crescentus* when compared with the WT. This seems to point to cells having a prolonged S-phase and thus not producing swarmer cells at the same rate as their WT counterparts.

### Cell Envelope Maintenance

Finally, we observe differential expression of proteins associated with the outer layer, specifically RfbB, ChvI, and RsaA. As previously mentioned, the expression of RsaA is dramatically reduced in the Δ*hnox* mutant strain at both the transcript and protein levels, resulting in a marked sensitivity to osmotic stress. As RsaA is constitutively expressed in WT *C. crescentus*, this implies a relationship between the H-NOX signaling pathway and RsaA expression. As what appears to be an attempt to adjust to less RsaA production, we observe a marked upregulation of RsaF_a_ and PerA, two proteins that lie within the same operon. PerA plays a role in production of GDP-*N*-acetylperosamine, a major component of the *C. crescentus* O-antigen (64). RsaF_a_, on the other hand, is an outer-membrane transporter of RsaA (65). RfbB and ChvI also exhibit an upregulation in the Δ*hnox* strain of *C. crescentus*. RfbB has been related to RsaA anchoring as well as synthesis of the lipopolysacharide within *C. crescentus* (66). ChvI is a response regulator that maintains cell envelope homeostasis through changes to the inner-membrane proteins, peptidoglycan usage, and adjusting the outer membrane. The ChvGI two-component signaling system has also been associated with osmotic shock responses (38). The upregulation of RfbB and PerA appears to be an attempt to anchor RsaA to the envelope, whereas ChvI seems to be a direct consequence of the osmotic stress resulting from the lack of RsaA. In addition, several other proteins pertaining to an osmotic stress response exhibit an increase in expression. As biofilm formation has been reported to be an outcome of stress response, there is a possibility that the increase of surface-attached biomass in Δ*hnox C. crescentus* could potentially be attributed to a defense mechanism. Finally, the reported growth inhibition when plated on KCl and decrease in cell size appear to coincide with the Δ*hnox* strain of *C. crescentus* exhibiting increased osmotic stress.

### Remaining Questions

Taken together, the phenotypic and proteome data described previously suggest a key role for H-NOX in cell cycle control and surface layer production in *C. crescentus*. However, the mechanism by which H-NOX exhibits its effects is still unclear. C-di-GMP is a major determinant of progression to S-phase (12), and it is significantly increased in the mutant strain. H-NOX may regulate HnoK, which may phosphorylate DGC and/or PDE enzymes, modulating their activity and altering c-di-GMP pools as has been observed in other organisms (32, 33, 67). This would not alter the expression of these enzymes, and indeed, we see no differential expression of any protein annotated as DGC or PDE. The precise targets of HnoK phosphorylation and how H-NOX regulates this process will be greatly aided by phosphoproteome data and phosphotransfer profiling experiments, both of which are underway in our laboratory.

We are also interested in the dramatic impact of H-NOX on RsaA expression. The *rsaA* promoter is described throughout the literature as constitutive (68, 69, 70). To our knowledge, no transcription factors are known to influence its expression. Our data suggest that H-NOX signaling involves such a transcription factor, possibly controlled by phosphorylation, to maintain high levels of RsaA expression throughout the cell cycle. Here again, identification of this protein will likely be aided by phosphoproteome experiments.

Finally, although H-NOX is described as an NO sensor, the role of NO in *C. crescentus* H-NOX is ambiguous. Addition of low concentrations of NO to WT *C. crescentus* cultures did not significantly influence biofilm formation, nor did we observe a differential effect of NO on WT *versus* Δ*hnox* strains (data not shown). Clearly, NO is not necessary for H-NOX signaling in this species. Furthermore, it is unlikely that *C. crescentus* would regularly encounter NO in its native environment of pristine fresh water. It may be that H-NOX is a constitutive regulator in *C. crescentus*, or it may respond to environmental signals different from, or in addition to, NO. It may itself be regulated by the cell cycle. *In vitro* experiments on how the H-NOX heme state influences regulation of HnoK activity and temporal expression studies will help to solve these open questions.

## Data Availability

MS data have been deposited to the ProteomeXchange Consortium *via* the PRIDE partner repository with the dataset identifier MSV000092147, and the annotated MS/MS spectra can be accessed using the search key “fliluwphjk” in https://msviewer.ucsf.edu/prospector/cgi-bin/msform.cgi?form=msviewer. All other data are contained within the article, the supplemental data, or available upon request from the authors.

## Supplemental data

This article contains supplemental data.

## Conflict of interest

The authors declare no competing interests.
