# Supplementary material for: Influence of the Heme Nitric Oxide/Oxygen Binding Protein (H-NOX) on Cell Cycle Regulation in Caulobacter crescentus
Source: Mol Cell Proteomics. 2023 Nov 17;22(12):100679. doi: 10.1016/j.mcpro.2023.100679 (PMC10746521; doi:10.1016/j.mcpro.2023.100679)
Supplement: Supplemental figures [file mmc3.docx]

**SUPPLEMENTAL FIGURES**

**
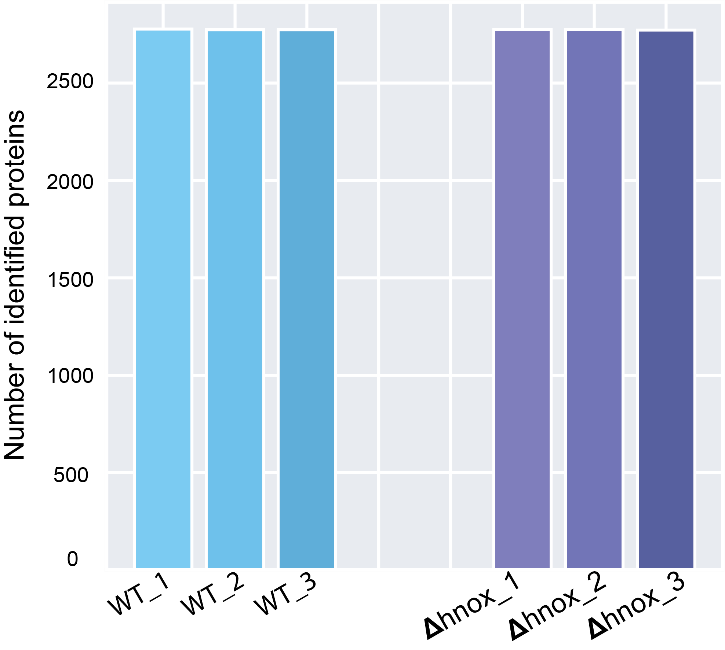
**

**Supplemental figure 1:** Protein identification in Caulobacter. The bar diagram illustrates the quantification of proteins identified in the two strains *Δhnox* and WT *C. crescentus*.

**
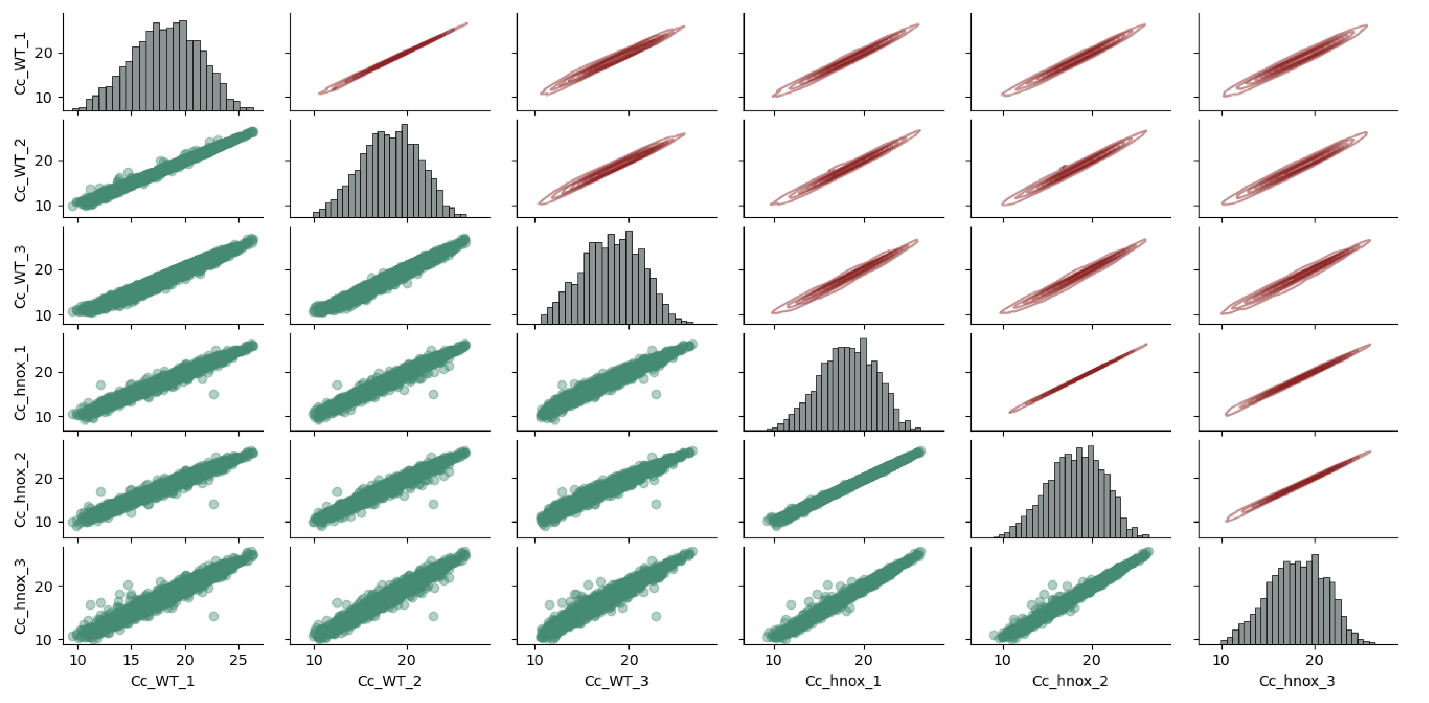
**

**Supplemental figure 2:** The multi plotting represents the logarithmic protein ratios of all samples against one another. The diagonal of the figure displays the histograms for each individual sample. Above the diagonal, the 2D kernel density estimates of the samples were depicted in relation to one another

**
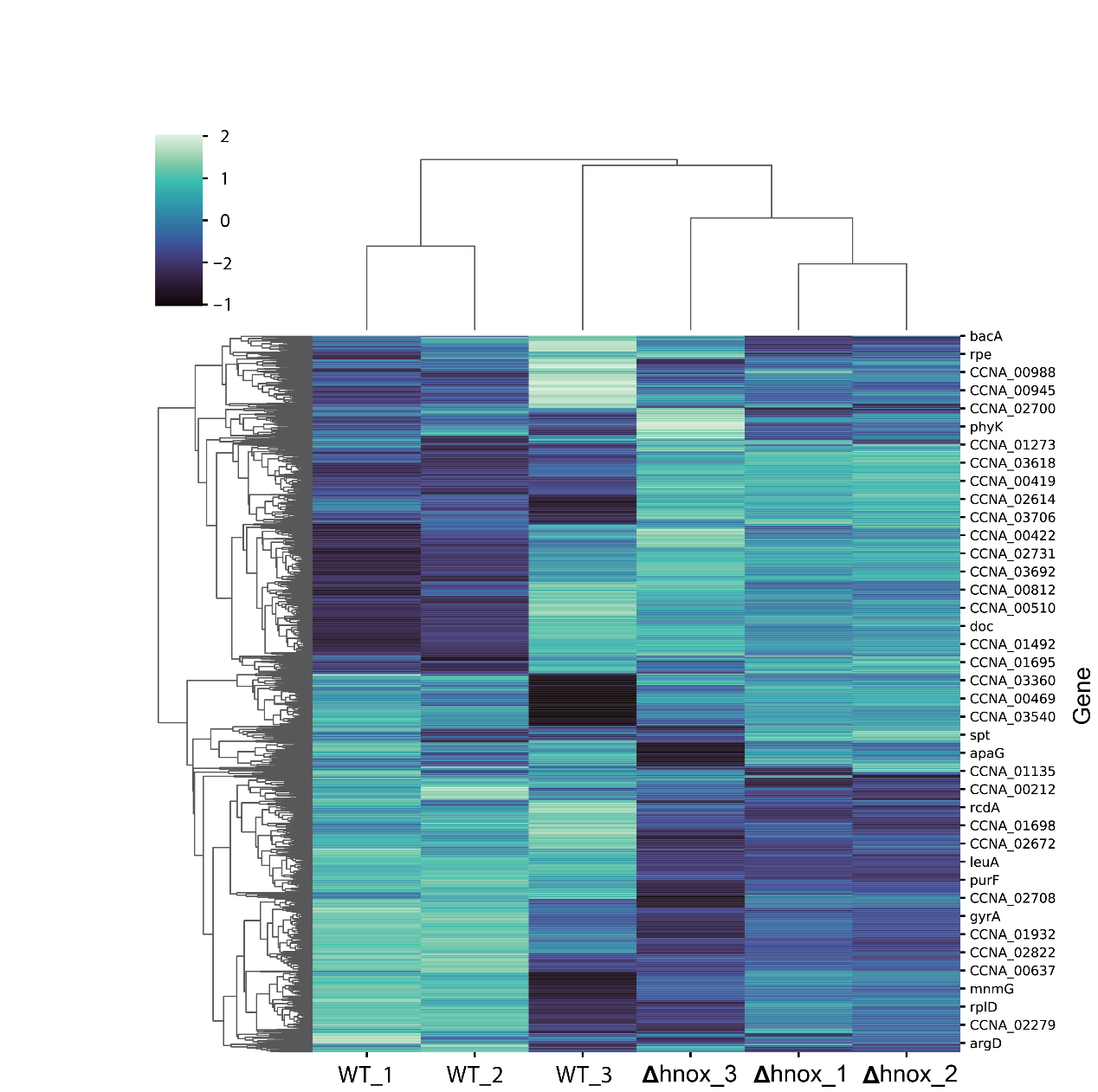
**

**Supplemental figure 3:** Hierarchical clustering of quantified proteins. Certain protein clusters exhibit elevated (turquoise) or reduced (slateblue) expression levels in Δhnox in comparison to WT strains.
